# Allogeneic cardiosphere-derived cells (CAP-1002) in critically ill COVID-19 patients: compassionate-use case series

**DOI:** 10.1007/s00395-020-0795-1

**Published:** 2020-05-12

**Authors:** Siddharth Singh, Tarun Chakravarty, Peter Chen, Akbarshakh Akhmerov, Jeremy Falk, Oren Friedman, Tanzira Zaman, Joseph E. Ebinger, Mitch Gheorghiu, Linda Marbán, Eduardo Marbán, Raj R. Makkar

**Affiliations:** 1 Cedars-Sinai Medical Center, Smidt Heart Institute, 127 S. San Vicente Boulevard, Advanced Health Sciences Pavilion, Third Floor, Suite A3100, Los Angeles, CA 90048 USA; 2 Division of Pulmonary and Critical Care Medicine, Department of Medicine, Cedars-Sinai Medical Center, Los Angeles, CA 90048 USA; 3 Capricor Inc, Beverly Hills, CA 90211 USA

**Keywords:** Coronavirus, Hyperinflammation, Cytokine storm, Cell therapy

## Abstract

There are no definitive therapies for patients with severe acute respiratory syndrome coronavirus-2 (SARS-CoV-2) infection. Therefore, new therapeutic strategies are needed to improve clinical outcomes, particularly in patients with severe disease. This case series explores the safety and effectiveness of intravenous allogeneic cardiosphere-derived cells (CDCs), formulated as CAP-1002, in critically ill patients with confirmed coronavirus disease 2019 (COVID-19). Adverse reactions to CAP-1002, clinical status on the World Health Organization (WHO) ordinal scale, and changes in pro-inflammatory biomarkers and leukocyte counts were analyzed. All patients (*n* = 6; age range 19–75 years, 1 female) required ventilatory support (invasive mechanical ventilation, *n* = 5) with PaO_2_/FiO_2_ ranging from 69 to 198. No adverse events related to CAP-1002 administration were observed. Four patients (67%) were weaned from respiratory support and discharged from the hospital. One patient remains mechanically ventilated as of April 28th, 2020; all survive. A contemporaneous control group of critically ill COVID-19 patients (*n* = 34) at our institution showed 18% overall mortality at a similar stage of hospitalization. Ferritin was elevated in all patients at baseline (range of all patients 605.43–2991.52 ng/ml) and decreased in 5/6 patients (range of all patients 252.89–1029.90 ng/ml). Absolute lymphocyte counts were low in 5/6 patients at baseline (range 0.26–0.82 × 10^3^/µl) but had increased in three of these five patients at last follow-up (range 0.23–1.02 × 10^3^/µl). In this series of six critically ill COVID-19 patients, intravenous infusion of CAP-1002 was well tolerated and associated with resolution of critical illness in 4 patients. This series demonstrates the apparent safety of CAP-1002 in COVID-19. While this initial experience is promising, efficacy will need to be further assessed in a randomized controlled trial.

## Introduction

On April 28, 2020, the number of confirmed patients with SARS CoV-2 infection (COVID-19) reached ~ 3 million worldwide, with > 200,000 deaths [45]. Although most cases are mild to moderate in severity, about 15% develop severe pneumonia, and nearly 5% progress to acute respiratory distress syndrome and multiple organ failure [25, 49]. This worsening is predominantly driven by cytokine upregulation and an exaggerated yet maladaptive inflammatory response [35, 40, 47]. Direct viral infection and cytopathic effects may also play a role. In the sickest patients, a hyperimmune response characterized by cytokine storm leads to critical illness and end-organ dysfunction, with high mortality. Mortality rates increase with severity of illness, and rates > 50% have been described in critically ill patients [7, 18, 48]. No measures, other than supportive therapy, have been found to be effective in treating COVID-19, constituting a major unmet medical need.

Immunomodulatory therapies under active investigation for COVID-19 include corticosteroids, anti-cytokine therapies (including monoclonal antibodies), convalescent plasma, intravenous immune globulin (IVIG) therapy, and inhibitors of specific inflammatory pathways, including Notch signaling [1, 38]. All treatments await validation in large-scale randomized controlled trials. Given their anti-inflammatory and immune-modulating effects, various cell types have also emerged as therapeutic candidates. In 2013–2014, 17 patients with H7N9 influenza were treated with mesenchymal stem cells (MSCs) and outcomes were compared to 44 controls. Higher survival rate was noted in the MSC group compared to controls (82.4% vs 45.5%, respectively) [12]. For COVID-19, MSCs were recently investigated in a small case series out of China, but only one patient was critically ill [26].

**Fig. 1:**
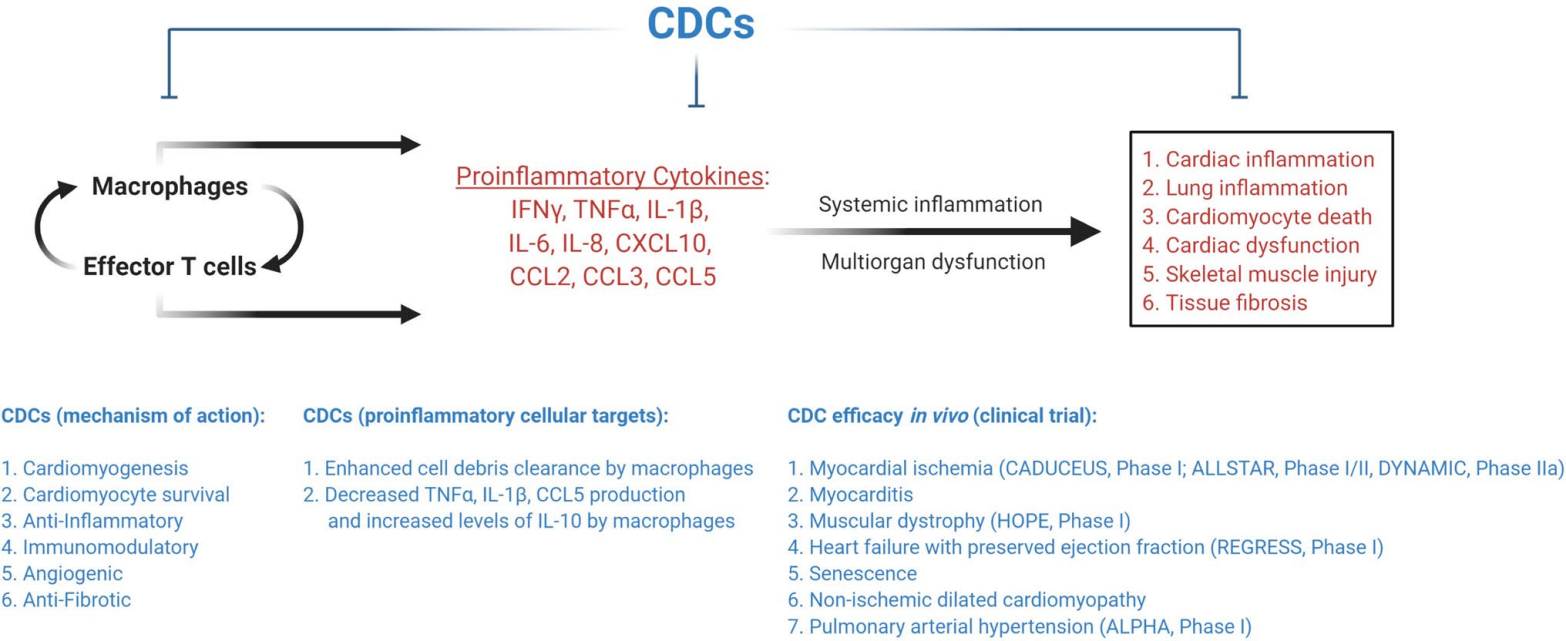
CDC-sensitive targets in COVID-19 pathogenesis. CDC-sensitive targets in COVID-19 pathogenesis involve modulation of macrophages, effector T cells, and pro-inflammatory cytokines. Key citations can be found in Marbán, E. Nat Biomed Eng 2018 and de Couto, G. Exp Mol Med 2019 [15, 30]

Cardiosphere-derived cells (CDCs) are stromal/progenitor cells, derived from heart tissue, with a distinctive antigenic profile (CD105^+^, CD45^−^, CD90^low^) [13, 41]. These cells are entirely distinct from c-kit^+^ putative cardiac progenitors [13, 27], which have been the subject of various retracted studies [23]. Since CDCs were first isolated from human endomyocardial biopsies in 2007, these cells have been tested in > 200 patients in clinical trials for myocardial infarction, heart failure with reduced and preserved ejection fraction, Duchenne muscular dystrophy, pulmonary arterial hypertension, and hypoplastic left heart syndrome [29, 30]. These trials, along with extensive preclinical investigation (~ 200 publications from > 55 independent laboratories worldwide), have demonstrated immunomodulatory and anti-inflammatory effects of CDCs (Fig. 1) [30]. Head-to-head comparisons in preclinical models indicate that CDCs may be more effective than MSCs with regards to paracrine factor secretion and myocardial remodeling [27]. Given the safety record of CDCs in humans, and the substantial body of evidence confirming relevant disease-modifying bioactivity, applicability to COVID-19 seemed compelling, particularly in the hyperinflammatory stage of the illness. Due to the scope of the pandemic and unmet need for effective therapies, we evaluated safety and impact of administration of allogeneic CDCs, formulated for intravenous (IV) infusion as CAP-1002, in critically ill COVID-19 patients.

## Methods

The study was conducted at Cedars-Sinai Medical Center (CSMC), Los Angeles, California, USA from March 25, 2020 to the final date of follow-up on 4/28/2020. Each individual case study was approved by the Institutional Review Board at CSMC, and each patient (or their legal representative) gave written informed consent. Likewise, each case was reviewed in detail with the US Food and Drug Administration (FDA) to obtain approval for compassionate administration of CAP-1002 under Emergency Use protocols.

### Patients

Patients were evaluated for possible recruitment if they met the following criteria: (1) laboratory confirmed COVID-19, diagnosed using a reverse transcriptase polymerase chain reaction (RT-PCR) assay; (2) severe COVID-19, with respiratory failure requiring increasing supplemental oxygen and/or shock requiring inotropes; (3) not enrolled in another clinical trial of an experimental agent for COVID-19; and (4) ability of the patient (or legally authorized representative) to provide informed consent. Patients received adjunctive therapy (including hydroxychloroquine and tocilizumab) per clinical practice protocols then in place for COVID-19 at CSMC. Lack of clinical improvement or deterioration despite standard care was the primary reason to evaluate patients for emergent administration of allogeneic CDCs. Exclusion criteria included known hypersensitivity to dimethyl sulfoxide (DMSO; a component of CAP-1002), prior stem cell therapy, pre-existing terminal illness (e.g., metastatic cancer), need for mechanical circulatory support and dialysis. In general, patients with multi-organ failure who were deemed to be too sick for any intervention were excluded from the study.

### Contemporaneous control group

Given the compassionate use nature of the case series, there was no randomization. Nevertheless, we sought some basis for comparison of outcomes and clinical characteristics between the 6 CAP-1002-treated subjects reported here, and other critically ill patients simultaneously hospitalized for COVID-19 at our institution, with similar baseline characteristics. We, therefore, retrospectively characterized a set of patients admitted to CSMC on or after 3/1/2020 with RT-PCR confirmed SARS-CoV-2 infection, who required mechanical ventilation. To render the comparison as fair as possible, we quantified clinical status at 30.7 days of hospitalization (to match the average in the CAP-1002-treated subjects) and limited the analysis to patients who received anti-IL6 or anti-IL6 receptor agents during the hospitalization as standard-of-care therapy (as did all of the CAP-1002-treated subjects). Patients were excluded if they: (1) did not have at least 30.7 days of follow-up from admission to the terminal event (death or hospital discharge); (2) were enrolled in any clinical trial requiring informed consent; and (3) had a tracheostomy placed prior to the current admission.

### Cell manufacturing

CAP-1002 was manufactured by Capricor using reported methods [11, 41]. Based on the IV infusion protocol and dose of CAP-1002 used in the HOPE-2 clinical trial of Duchenne muscular dystrophy (NCT03406780), we selected a dose of 150 million allogeneic CDCs to administer in this study. Cell product frozen concentrate was supplied in a volume of 10 ml of cryogenic cell preservation solution consisting of 50% (v/v) CryoStor® CS10 containing 10% DMSO, 40% (v/v) HypoThermosol®, and 10% (v/v) Albumin (Human, HSA) 25%. The primary container was a Daikyo Crystal Zenith® vial with chlorobutyl stopper that was stored at less than or equal to -140 ˚C until thawed for use. Once thawed, each vial of CAP-1002 was diluted with 40 ml of 5% HSA for IV use only, containing the albumin component of human blood in a 60-ml syringe.

### Cell administration

CAP-1002 was administered IV via a peripheral access device (or a central venous access device, if it was available at the time of administration). Following administration of the cells, the patients were monitored for signs of clinical and biochemical improvement (or deterioration). If the patients continued to require significant supplemental oxygen after one week, they were re-evaluated for a second dose of cell administration as a booster dose. Prior to administration of the second dose, patients were premedicated with an antihistamine to mitigate any potential allergic reactions (which, though clinically insignificant, were noted in two patients in the HOPE-2 clinical trial when premedication was omitted before the second dose). The rationale for a second administration of CAP-1002 is based on preclinical evidence demonstrating additional benefit with repeat dosing of CDCs. Importantly, a repeat administration of CDCs was not associated with sensitization or adverse immunologic responses in rodents [2, 36].

The final ready-to-use cell product in a total volume of 50 ml was infused with a commercially available syringe pump [510(k) cleared for human use] set at an initial rate of 1 ml/min for 10 min (± 30 s). If no signs or symptoms of a potential adverse reaction were observed, the infusion was continued at a rate of 4 ml/min for a total infusion time of 20 min (± 2 min). Upon completion of infusion of the first vial of CAP-1002, and prior to infusion of the second vial, residual material was washed out of the IV tubing with a bolus of 5% HSA (approximately 4 ml over 2 min ± 30 s). The next cell dose of 75 million cells in the second 60-ml syringe was administered to the subject at a rate of 4 ml/min, for a total infusion time of 12.5 (± 1 min). After the second infusion, residual material was washed out of the IV tubing with a bolus of 5% HSA (approximately 4 ml over 2 min ± 30 s).

### RT-PCR

RT-PCR was performed on nasopharyngeal specimens collected from patients who met Centers for Disease Control and Prevention clinical and/or epidemiological criteria for COVID-19 testing [10]. If RNA from SARS-CoV-2 was detected, the test was positive and the patient was considered infected with virus and presumed to be contagious. Laboratory test results were considered in the context of clinical observations and epidemiologic data in making a final diagnosis and patient management decisions. The test was developed and its performance characteristics were determined by CSMC Department of Pathology and Laboratory Medicine. The laboratory is certified under the Clinical Laboratory Improvement Amendments (CLIA) as qualified to perform high-complexity clinical laboratory testing. This test was validated, but independent review by FDA of this validation is pending.

### Statistical analysis

Pooled data are presented as means ± standard deviation (SD), range, and median. Due to small sample size, statistical tests comparing CAP-1002 and control groups were not performed.

## Results

Six patients (age range 19–75 years, one woman) underwent IV infusion of CAP-1002 containing 150 million allogeneic CDCs. Consistent with the Berlin Criteria, all patients had acute respiratory distress syndrome (ARDS) prior to CAP-1002 infusion, with decreased PaO_2_/FiO_2_ ratios (range 69.0–198.0; median 142.5), diffuse bilateral pulmonary infiltrates on chest imaging (*n* = 6, 100%), and evidence of preserved cardiac function on transthoracic echocardiography (LVEF range 50–75%; median 55%), suggesting a non-cardiac origin of respiratory failure [4]. Sequential organ failure and assessment (SOFA) scores ranged from 2 to 8 prior to cell administration [19]. All six patients had received an anti-IL6 agent, tocilizumab, prior to the CAP-1002 infusion. In addition to tocilizumab, patient 1 received lopinavir/ritonavir and the remaining patients received hydroxychloroquine for 5 days prior to the cell infusion. Patient 2 was 19 years old, but was morbidly obese (BMI 42.2). Patients 1, 3, 4, 5 had significant cardiovascular comorbidities (Table 1). All patients were intubated (*n* = 5) or required high-flow nasal cannula (HFNC) support (*n* = 1) at the time of enrollment. The one patient receiving HFNC support was deemed high risk for imminent intubation. The remaining intubated patients had been on invasive mechanical ventilation for 2–11 days prior to cell administration. The first patient was intubated at the time of enrollment, but extubated by the time of cell administration. This patient was determined to be at a high risk for reintubation given high oxygen requirements on HFNC and, therefore, underwent cell administration.

**Table 1:** Clinical characteristics of critically ill patients with COVID-19

Variable	Patient 1	Patient 2	Patient 3	Patient 4	Patient 5	Patient 6
Age (years)	75	19	55	71	60	58
Gender	Male	Male	Male	Female	Male	Male
Weight (kg)	75.3	133.4	104.3	58.9	82.4	62.6
BMI (kg/m^2^)	23.2	42.2	33.6	26.4	32.2	22.3
Smoking	No	No	No	No	No	No
Prior comorbidities	Afib, HTN, HLD, T2DM	Obesity	HTN, HLD, HFpEF, T2DM, Obesity	T2DM, HLD, osteoporosis	CKD, HTN, obesity	None
Clinical presentation	Fever, chills, myalgia, cough, dyspnea, diarrhea	Fevers, chills, diarrhea, cough	Malaise, ADHF (edema, weight gain)	Fever, chills, SOB, cough, emesis	Fever, cough, dyspnea, diarrhea, emesis	Fevers, cough, SOB, diarrhea
Time from symptom onset to admission (days)	7	4	7	7	8	9
Days hospitalized (days)	26	16	32	28 (ongoing)	14	8 (ongoing)
Time from admission to critical care transfer (days)	3	3	9	2	0	1
Days from ICU admission to first infusion	12	5	7	8	3	2
Duration of MV before CDC (days)	11^a^	5	6	8	2	–^b^
Days from infusion to extubation	–^a^	3	4	Patient is still intubated, as of 04/28/2020	1	–^b^
Total MV duration (days)	11	8	10	Ongoing	3	–^b^
Pa/FiO_2_ prior to infusion (P/F)	P/F: 69	P/F: 145	P/F: 173	P/F: 140	P/F: 198	P/F: 93
SOFA prior to infusion	3	4	5	8	5	2
Prior treatments	Lopinavir-ritonavir (× 5 days) Tocilizumab (× 1 dose)	HCQ (× 5 days) Tocilizumab (× 1 dose)	HCQ (× 5 days) Tocilizumab (× 1 dose)	HCQ (× 5 days) Tocilizumab (× 1 dose)	HCQ (× 5 days) Tocilizumab (× 1 dose)	HCQ (× 5 days) Tocilizumab (× 1 dose)
Status	Discharged, alive	Discharged, alive	Discharged, alive	ICU, alive	Discharged, alive	ICU, alive

*ADHF* acute decompensated heart failure, *ARDS* acute respiratory distress syndrome, *CKD* chronic kidney disease, *HCQ* hydroxychloroquine, *HLD* hyperlipidemia, *HTN* hypertension, *HFpEF* heart failure with preserved ejection fraction, *ICU* intensive care unit, *MV* mechanical ventilation, *SOB* shortness of breath, *T2DM* Type 2 diabetes mellitus

^a^Patient received first dose shortly after extubation due to rising oxygen requirements and imminent reintubation

^b^Patient required high-flow nasal cannula support and received CAP-1002 to prevent invasive mechanical ventilation

**Fig. 2:**
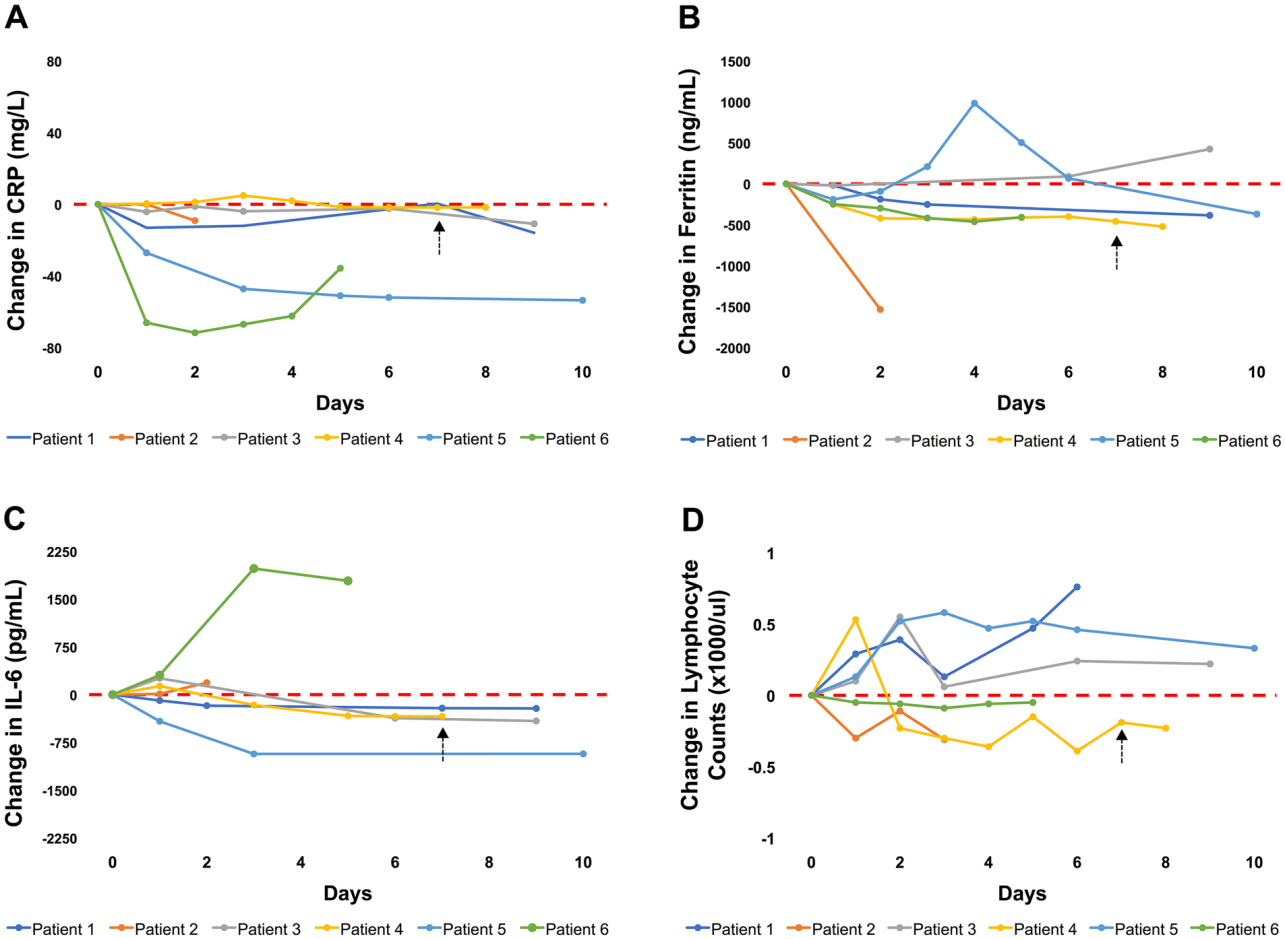
Trends in inflammatory markers and lymphocyte counts. Changes in the level of inflammatory markers, including C-reactive protein (**a**), ferritin (**b**), IL-6 (**c**), and lymphocyte counts (**d**) within 10 days of CAP-1002 infusion, normalized to baseline values (pre-infusion). Dashed arrows indicated the time of second infusion

Blood cell counts, inflammatory markers and cytokine levels are summarized in Table 2 and Fig. 2. All patients had elevated CRP (Fig. 2a), ferritin (Fig. 2b), IL-6 (Fig. 2c) and TNFα prior to cell infusion. Five patients had lymphopenia prior to infusion (Fig. 2d). IL1α and IL-1β levels were not elevated in this cohort. Ferritin and CRP levels decreased in 5 out of 6 patients following cell infusion (Fig. 2b and a, respectively). IL-6 levels were increased in all six patients at baseline and decreased in four patients (Fig. 2c). IL-10 levels remained below reference range in one patient, decreased in three patients, and increased in two patients (Table 2). Upon admission, two patients had mildly elevated cardiac troponin I levels (range of all patients < 0.02–0.07 ng/ml; median 0.01 ng/ml). During the course of the hospitalization, cardiac troponin I levels increased in 4 patients (range of all patients < 0.02–1.26 ng/ml; median 0.13 ng/ml) within 4–16 days of admission, but subsequently decreased in all these patients (range of all patients < 0.02–0.15 ng/ml; median 0.07 ng/ml). Similarly, d-dimer levels were mildly elevated in four patients upon admission (range of all patients 0.34–2.22 μg/ml; median 0.83 μg/ml), increased in five patients within 4–17 days of admission (range of all patients 5.36–20.00 μg/ml; median 20.00 μg/ml), and subsequently decreased in four patients (range of all patients 1.53–20.00 μg/ml; median 2.45 μg/ml).

**Table 2:** Leukocyte counts and inflammatory markers in patients receiving CAP-1002

	Patient 1	Patient 2	Patient 3	Patient 4	Patient 5	Patient 6
WBC (× 1000/ul)	Day 0: 12.87 Day 1: 13.84 Day 2: 12,99 Day 3: 12.85 Day 4: 10.01 Day 5: 8.08 Day 6: 5.64 Day 7: 6.79	Day 0: 5.8 Day 1: 8.4 Day 2: 6.39 Day 3: 9.86 Day 5: 8.02 Day 7: 5.63	Day 0: 10.17 Day 1: 10.35 Day 2: 8.63 Day 3: 9.34 Day 6: 7.50 Day 9: 4.34	Day 0: 13.79 Day 1: 13.50 Day 2: 11.76 Day 4: 13.07 Day 5: 11.59 Day 6: 14.51(pre 2nd dose) Day 7: 15.67 (post 2nd dose) Day 8: 14.67 Day 18: 23.13	Day 0: 6.38 Day 1: 7.24 Day 2: 9.79 Day 3 10.45 Day 4: 9.75 Day 5: 8.15 Day 6: 7.2 Day 10: 4.69	Day 0: 4.41 Day 1: 8.32 Day 2: 7.84 Day 3: 9.03 Day 4: 10.78 Day 5: 13.69
Lymphocytes (× 1000/ul)	Day 0: 0.26 Day 1: 0.55 Day 2: 0.65 Day 3: 0.39 Day 5: 0.73 Day 6: 1.02	Day 0: 1.40 Day 1: 1.1 Day 2: 1.29 Day 3 1.09	Day 0: 0.31 Day 1: 0.41 Day 2: 0.86 Day 3: 0.37 Day 6: 0.55 Day 9: 0.53	Day 0: 0.82 Day 1: 1.35 Day 2: 0.59 Day 3: 0.52 Day 4: 0.46 Day 5: 0.67 Day 6: 0. 43 (pre 2nd dose) Day 7: 0.63 (post 2nd dose) Day 8: 0.59 Day 18: 0.23	Day 0: 0.33 Day 1: 0.46 Day 2: 0.85 Day 3: 0.91 Day 4: 0.80 Day 5: 0.85 Day 6: 0.79 Day 10: 0.66	Day 0: 0.54 Day 1: 0.49 Day 2: 0.48 Day 3: 0.45 Day 4: 0.48 Day 5: 0.49
C-reactive protein (mg/l)	Day 0: 31.9 Day 1: 18.8 Day 3: 19.9 Day 7: 32.3 (pre 2nd dose) Day 9: 16.0 (post 2nd dose)	Day 0: 15.2 Day 2: 6.1	Day 0: 19.4 Day 1: 15.2 Day 2: 18.2 Day 3: 15.5 Day 6: 17.0 Day 9: 8.5	Day 0: 2.0 Day 1: 2.4 Day 2: 3.2 Day 3: 6.9 Day 4: 4.0 Day 5: 0.5 Day 6: 0.3 (pre 2nd dose) Day 7: 0.2 (post 2nd dose) Day 8: 0.3 Day 18: 54.6	Day 0: 54.6 Day 1: 27.5 Day 3: 7.4 Day 5: 3.6 Day 6: 2.6 Day 10: 1.0	Day 0: 80.3 Day 1: 14.2 Day 2: 8.6 Day 3: 13.3 Day 4: 17.9 Day 5: 44.6
Ferritin (ng/ml)	Day 0: 774.52 Day 1: 755 Day 2: 586.26 Day 7: 522.76 (pre 2nd dose) Day 9: 389.45 (post 2nd dose)	Day 0: 2991.52 Day 2: 1457.84	Day 0: 605.43 Day 1: 584.26 Day 6: 695.76 Day 9: 1029.90	Day 0: 866.81 Day 1: 615.84 Day 2: 445.95 Day 4: 432.93 Day 5: 455.5 Day 6: 466.05 (pre 2nd dose) Day 7: 408.5 (post 2nd dose) Day 8: 344.7 Day 11: 252.89	Day 0: 1281.97 Day 1: 1090.98 Day 2: 1,189.54 Day 3: 1491.9 Day 4: 2266.0 Day 5: 1785.3 Day 6: 1349.8 Day 10: 912.55	Day 0: 1096.83 Day 1: 849.10 Day 2: 798.58 Day 3: 679.62 Day 4: 634.50 Day 5: 688.51
IL-6 (pg/ml)	Day 0: 216.7 Day 1: 124.3 Day 2: 44.2 Day 7: 4.0 (pre 2nd dose) Day 9: < 3.2 (post 2nd dose)	Day 0: 79.8 Day 1: 90.7 Day 2: 267.2	Day 0: 421.5 Day 1: 678.6 Day 6: 52.7 Day 9: 7.9	Day 0: 348.3 Day 1: 482.3 Day 3: 185.2 Day 5: 14.9 Day 6: 7.1 (pre 2nd dose) Day 7: 8 (post 2nd dose) Day 11: < 3.2	Day 0: 1,006.9 Day 1: 588.7 Day 3: 75.7 Day 5: 102.4 Day 10: 77.7	Day 0: 594.7 Day 1:900.5 Day 3:2573.9 Day 5: 2381.4
IL-1α (pg/ml)	Day 0: < 3.9 Day 1: < 3.9 Day 2: < 3.9	Day 0: < 3.9 Day 2: < 3.9	Day 0: – Day 1: – Day 6: –	Day 0: < 3.9 Day 1: < 3.9 Day 3: < 3.9 Day 5: < 3.9	Day 0: < 3.9 Day 1: < 3.9 Day 3: < 3.9	Day 0: – Day 1: – Day 3: – Day 5: –
IL-1β (pg/ml)	Day 0: < 3.2 Day 1: 4.5 Day 2: < 3.2 Day 7: -NA Day 9: -NA	Day 0: < 3.2 Day 1: < 3.2 Day 2: < 3.2	Day 0: < 3.2 Day 1: < 3.2 Day 6: < 3.2	Day 0: < 3.2 Day 1: < 3.2 Day 3: < 3.2 Day 5: < 3.2	Day 0: < 3.2 Day 1: < 3.2 Day 3: < 3.2 Day 5: < 3.2	Day 0: < 3.2 Day 1: < 3.2 Day 3: < 3.2 Day 5: < 3.2
TNF-α (pg/ml)	Day 0: 7.0 Day 1: 4.5 Day 2: 13.1	Day 0: 14.6 Day 1: – Day 2: 30.1	Day 0: 46.7 Day 1: 30.5 Day 6: 24.3	Day 0: 52.5 Day 1: 55.6 Day 3: 35.9 Day 5: 13.6 Day 7: 29.2 (post 2nd dose) Day 11: 19.6	Day 0: 15.6 Day 1: 20.4 Day 3: 22.8 Day 5: 25.2 Day 10: 16.0	Day 0: 17.9 Day 1: – Day 3: 49.4 Day 5: 22.9
IL-10 (pg/ml)	Day 0: < 3.2 Day 1: < 3.2 Day 2: < 3.2 Day 7: < 3.2 (pre 2nd dose) Day 9: < 3.2 (post 2nd dose)	Day 0: 6.1 Day 1: 3.5 Day 2: 10.3	Day 0: 7.5 Day 1: 4.8 Day 6: < 3.2 Day 9: < 3.2	Day 0: 6.3 Day 1: 7.0 Day 3: 3.3 Day 5: < 3.2 Day 7: 10.2 (post 2nd dose) Day 11: < 3.2	Day 0: 8.6 Day 1: 14.8 Day 3: 9.5 Day 5: 8	Day 0: 11.6 Day 1: 15.1 Day 3: 16.7 Day 5: 14.8

Day 0 shows laboratory values prior to the first administration of CAP-1002. Patients 1 and 4 received a second dose of CAP-1002, 7 days after the first infusion

**Fig. 3:**
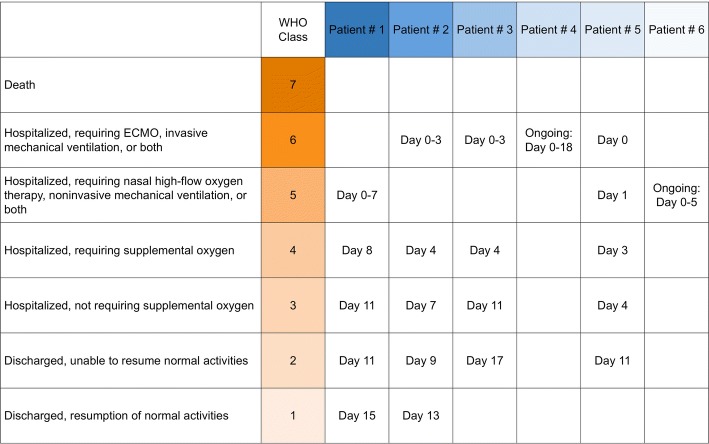
Clinical course of patients receiving CAP-1002. The clinical course is plotted for individual patients on the 7-point WHO scale for evaluation of patients with COVID-19. Day 0 denotes time of CAP-1002 infusion

The clinical course of individual patients is summarized in Fig. 3. No patient experienced any complications related to CAP-1002 administration. Patients 1 and 4 received a second dose of CAP-1002, after pretreatment with diphenhydramine, likewise with no complications. All intubated patients improved clinically after cell infusion, except patients 4 and 6, who are still in ICU but remain stable. Patient 1 was already extubated at the time of infusion, and CAP-1002 was administered due to rising oxygen requirements (on HFNC) and concern for reintubation. Similarly, patient 6 received the infusion in the setting of respiratory deterioration and imminent risk for intubation. As of April 28, 2020 (5 days post-infusion), patient 6 has not been intubated. Patients 2, 3 and 5 were extubated on post-infusion days 3, 4 and 1, respectively. Except for patients 4 and 6, all patients were transferred out of the ICU and discharged from the hospital (Table 1). There were no overt instances of acute stroke, acute myocardial infarction, or acute pulmonary embolism during the hospitalization at the time of last follow-up. One patient (patient #4) received additional antibiotics for a possible bacterial superinfection, although the diagnosis remains presumptive and no source has been identified. All patients are alive as of April 28, 2020, with a mean follow-up of 13.5 ± 4.6 days. This is in contrast to 6 deaths (18%) noted among 34 similar patients who received treatment for COVID-19 in our medical ICU around the same time (notably all patients were treated with anti-IL6 therapy; Table 3).

**Table 3:** Outcomes and characteristics of CAP-1002-treated COVID-19 patients and a contemporaneous control group

	Contemporaneous Controls^a^				CAP-1002		
	All	Discharged alive	Remain hospitalized	Dead	All	Discharged alive	Remain hospitalized
	*N* = 34	*N* = 20	*N* = 8	*N* = 6	*N* = 6	*N* = 4	*N* = 2
Age, years	66.8 ± 13.6	66.9 ± 15.0	66.1 ± 10.3	67.3 ± 14.8	56.3 ± 19.9	52.3 ± 20.6	58 years, 71 years
Sex, male	26 (76)	13 (65)	8 (100)	5 (83)	5 (83)	4 (100)	1 (50)
Race							
White	18 (53)	13 (62)	3 (34)	2 (33)	1 (17)	1 (25)	0 (0)
African American	11 (32)	4 (20)	4 (50)	3 (50)	2 (33)	2 (50)	0 (0)
Asian	0 (0)	0 (0)	0 (0)	0 (0)	0 (0)	0 (0)	0 (0)
Other/unknown	5 (15)	5 (25)	1 (13)	1 (17)	0 (0)	0 (0)	0 (0)
Hispanic	6 (18)	3 (15)	1 (13)	1 (17)	3 (50)	1 (25)	2 (100)
Obesity	6 (18)	4 (20)	2 (25)	0 (0)	3 (50)	3 (75)	0 (0)
Hypertension	18 (53)	10 (50)	5 (63)	3 (50)	3 (50)	3 (75)	0 (0)
Diabetes	8 (24)	3 (15)	4 (50)	1 (17)	2 (33)	2 (50)	0 (0)
Prior MI or HF	7 (21)	5 (25)	2 (25)	0 (0)	1 (17)	1 (25)	0 (0)
COPD/asthma	7 (21)	4 (20)	2 (25)	1 (17)	0 (0)	0 (0)	0 (0)

*COPD* chronic obstructive pulmonary disease, *COVID-19* coronavirus disease 2019, *HF* heart failure, *MI* myocardial infarction

^a^Population consists of patients admitted to CSMC and requiring mechanical ventilation on or after 3/1/2020 with confirmed COVID-19 infection. Patients were excluded if they: (1) did not have at least 30.7 days of follow-up from admission to the terminal event (death or hospital discharge), in order to match the follow-up duration in the CAP-1002 group; (2) were enrolled in a clinical trial requiring informed consent; (3) did not receive an IL-6 inhibitor; or (4) had a tracheostomy placed prior to the current admission. Due to small sample sizes, statistical tests for comparison were not performed. Categorical data presented as total count and percentage (%), and continuous data are presented as mean ± standard deviation (SD)

## Discussion

Administration of CAP-1002 as a compassionate therapy for patients with severe COVID-19 and significant comorbidities was safe, well tolerated without serious adverse events, and associated with clinical improvement, as evidenced by extubation (or prevention of intubation). All the critically ill patients who received CAP-1002 survived, and four out of six have been discharged. This is in contrast to high mortality rates (~ 50%) reported for critically ill patients with COVID-19 [5]. Within our institution, an age- and gender-matched retrospectively assembled cohort of COVID-19 patients also showed higher mortality (6 of 34 patients) compared to the compassionate-use series (0 of 6), but statistical comparisons were not attempted given the small number of CAP-1002-treated patients. Most patients receiving CAP-1002 also showed improvements in inflammatory markers, though to varying degrees. Similar to other COVID-19 cohorts, our patients exhibited elevated cardiac troponin I and D-dimer levels [37, 44]. These biomarkers, however, decreased in all but 1 of the patients at the date of last follow-up.

The underlying pathophysiology of COVID-19 involves a maladaptive immune response to SARS-CoV-2 infection with increased levels of IL-6, IL-10, IL-2 and TNFα produced by macrophages, and fewer CD4^+^ and CD8^+^ T cells, but no significant changes in B-cell counts [1, 9, 43]. The dysregulated immune function with cytokine storm leads to lung, heart, and other end-organ injury [22]. Extensive preclinical and some clinical studies suggest that cell therapy may attenuate inflammation [30].

CDCs are stromal progenitor cells isolated from human heart tissue through well-specified culture techniques and exert their effects in a paracrine manner by secreting exosomes (nanosized vesicles with bioactive payload) [16, 17, 31, 39]. CDCs target multiple cytokine pathways (e.g., TNFα, IFN-γ, IL-1β, IL-6) that are associated with disease progression and poor outcomes in COVID-19 (Fig. 1). For example, CDCs have the capacity to polarize macrophages toward an anti-inflammatory and healing phenotype [30]. These anti-inflammatory effects have been demonstrated in animal models of myocardial ischemia, myocarditis, muscular dystrophy, aging, heart failure with preserved ejection fraction, pulmonary arterial hypertension and dilated cardiomyopathy [3, 20, 21, 33, 34, 42]. Finally, based on preclinical work, a majority of IV CDCs are retained in the lungs [6]. Thus, there may be local benefits within the lung parenchyma, although this remains a conjecture.

A number of as-yet-unaddressed issues pertinent to use of cell therapy in SARS-CoV-2 infection need to be highlighted. These include the optimal sources of cells and dosing strategies. With respect to safety, no serious adverse events have been noted with administration of CAP-1002 or MSCs from various sources in patients. Another issue related to safety is the potential secretion of IL-6 by CDCs [32]. This potential source of IL-6, however, appears to be negligible, as multiple preclinical models demonstrated the opposite effect following CDC administration: significantly decreased serum levels of IL-6 after CDC infusion [21, 24]. Thus, future studies should logically focus on recognized endpoints including overall mortality, survival to hospital discharge and beyond, length of hospital stay and ventilator-free days, with proper randomization and rigorous controls. A composite ordinal scale reflecting improvement in COVID-19 disease state may trenchantly capture the key clinical outcomes (as recommended by the WHO R&D Blueprint) [46].

### Limitations

Our study has several limitations. The absence of a true, randomized control group and the small sample size prevent any meaningful inferences about cause–effect relationship between receipt of CAP-1002 and clinical improvement. As per the standard care protocols for critically ill COVID-19 patients at CSMC, the patients enrolled in the study were receiving concomitant anti-viral and anti-inflammatory therapies, which may have confounded the positive clinical outcomes and introduced heterogeneity into assessments of circulating biomarkers. Nevertheless, we are encouraged by the higher survival rates in the CAP-1002 group when compared against a contemporaneous cohort that similarly received anti-IL-6 therapy and hydroxychloroquine. Furthermore, two drugs that were administered in our cohort of 6 patients and could have a potential confounding effect (lopinavir/ritonavir and hydroxychloroquine) were recently demonstrated to have no efficacy in COVID-19 patients [8, 28]. Remdesivir was not used in any of the patients reported here. These observations, however, do not definitively eliminate potential confounding.

Despite these limitations, the encouraging clinical outcomes and biochemical responses in the COVID-19 patients treated with CAP-1002 warrant further investigation in prospective registries and randomized placebo-controlled clinical trials. As an immediate successor to the present compassionate-use study, **C**dc**S** for **C**ytokine **S**torm in **C**ovid **S**yndrome Trial: **(CS)**^**3**^ is an expanded access protocol that is now exploring clinical and biochemical effects of CAP-1002 administration in patients with severe COVID-19 infection, under an Investigational New Drug application awarded April 22, 2020 [14]. A randomized controlled trial evaluating CAP-1002 versus placebo for the treatment of severe COVID-19 is currently in the advanced planning stages.

## Conclusions

In this initial series of six patients with severe COVID-19 and associated lung injury, administration of CAP-1002 was safely tolerated, and followed by improvement in clinical status and reductions in selective pro-inflammatory biomarkers in most patients. The small sample size and lack of a true, randomized control arm prevent conclusive assessment of this treatment modality—larger expanded use assessments and, most importantly, randomized placebo-controlled trials will more definitively assess this therapy.

## Data Availability

All described data are included within the manuscript. Any additional data are available upon request.
